# AG1024, an IGF-1 receptor inhibitor, ameliorates renal injury in rats with diabetic nephropathy via the SOCS/JAK2/STAT pathway

**DOI:** 10.1515/med-2023-0683

**Published:** 2023-04-06

**Authors:** Jianhua Liu, Yun Zhang, Min Liu, Feng Shi, Bo Cheng

**Affiliations:** Department of Nephrology, The Sixth Hospital of Wuhan (Affiliated Hospital of Jianghan University), Wuhan 430015, Hubei, China; Department of Nephrology, The Sixth Hospital of Wuhan (Affiliated Hospital of Jianghan University), No. 168, Hong Kong Road, Jiang’an District, Wuhan 430015, Hubei, China

**Keywords:** diabetic nephropathy, IGF-1 receptor, AG1024, fibrosis, inflammation, SOCS

## Abstract

Insulin-like-growth factor-1 (IGF-1) is the ligand for insulin-like growth factor-1 receptor (IGF-1R), and the roles of IGF-1/IGF-1R in diabetic nephropathy (DN) are well-characterized previously. However, the biological functions of AG1024 (an IGF-1R inhibitor) in DN remain unknown. This study investigates the roles and related mechanisms of AG-1024 in DN. The experimental DN was established via intraperitoneal injection of streptozotocin, and STZ-induced diabetic rats were treated with AG1024 (20 mg/kg/day) for 8 weeks. The 24 h proteinuria, blood glucose level, serum creatinine, and blood urea nitrogen were measured for biochemical analyses. The increase in 24 h proteinuria, blood glucose level, serum creatinine, and blood urea of DN rats were conspicuously abated by AG1024. After biochemical analyses, the renal tissue specimens were collected, and as revealed by hematoxylin and eosin staining and Masson staining, AG-1024 mitigated typical renal damage and interstitial fibrosis in DN rats. Then, the anti-inflammatory effect of AG-1024 was assessed by western blotting and ELISA. Mechanistically, AG-1024 upregulated SOCS1 and SOCS3 expression and decreased phosphorylated JAK2, STAT1, and STAT3, as shown by western blotting. Collectively, AG-1024 (an IGF-1R inhibitor) ameliorates renal injury in experimental DN by attenuating renal inflammation and fibrosis via the SOCS/JAK2/STAT pathway.

## 1 Introduction

Diabetes is a metabolic disorder with frequent occurrence and death rates [1]. Diabetic nephropathy (DN) is one of the most common and severe complications in diabetes mellitus patients and is the leading cause of end-stage renal disease [2]. The high incidence of DN in diabetes patients is approximately 30–40% [3]. Currently, despite available agents, the management of DN is still challenging [4]. The current therapeutic options have limited efficacy and adverse effects. Meanwhile, DN incidence is still increasing gradually [5]. Therefore, there is an urgent need to explore DN pathogenesis and initiate novel therapeutic options for DN intervention.

Inflammation is an indispensable player in DN pathogenesis [6]. Interleukin (IL)-6, mononuclear chemotactic protein-1 (MCP-1), and IL-1β are the most-studied inflammatory cytokines associated with renal inflammation and upregulation of these cytokines has been suggested to be associated with DN progression [7]. The fibroblasts recruited by the continuous effects of inflammation finally lead to renal fibrosis [8]. Diabetic renal fibrosis is attributed to glomerulosclerosis and renal tubulointerstitial fibrosis induced by excessive deposition of extracellular matrix (ECM), including fibronectin and collagen IV, causing DN pathogenesis [9]. Accumulation of ECM can be induced by activation of the transforming growth factor-β (TGF-β) [10]. TGF-β, as reported, is highly expressed in diabetic models, and its inhibition can prevent glomerular enlargement and reduce fibrosis [11]. Collectively, attenuation of inflammation and fibrosis by reducing inflammatory cytokines, decreasing accumulation of ECM, and inactivation of TGF-β, is a promising option for DN treatment.

The Janus kinase (JAK)/signal transducers and activators of transcription (STAT) pathway are involved in inflammation, and the activated JAK/STAT signal can intensify glomerular mesangial cell proliferation and matrix expansion, causing DN pathogenesis [12]. The JAK/STAT cascade comprises a few principal components. JAK1-3 and TYK2 are identified in the JAK family, and the STAT family has seven members, including STAT1-4, 5A/B, and 6 [13]. The binding of ligands to their receptors triggers the JAK/STAT signal cascade and subsequently regulates the expression of target genes encoding cytokines, chemokines, adherence molecules, and inducible enzymes such as inducible nitric oxide synthase (iNOS) and cyclooxygenase 2 (COX-2) [14]. Previous findings suggest that the JAK/STAT pathway, especially the JAK2/STAT1/STAT3-dependent axis, contributes to high glucose-mediated renal cell responses, including enhanced expression of genes involved in leukocyte infiltration, cell growth, and fibrosis [15–18]. Moreover, diverse approaches, including JAK2 inhibition, STAT1 antisense oligonucleotides, and STAT3 gene knockdown, have been proven to counteract the harmful effects of JAK/STAT activation in cultured renal cells [17,19–21] and in the development of diabetes *in vivo* [18,21]. The JAK/STAT pathway is negatively regulated by various mechanisms including the suppressor of cytokine signaling (SOCS) proteins. SOCS family members (CIS; SOCS1-7), particularly SOCS1 and SOCS3, control the magnitude and duration of JAK/STAT signaling [22]. Studies have shown that overexpression of SOCS in the kidney can relieve the progression of DN by inhibiting the JAK/STAT pathway [23,24]. Therefore, this study focuses on the prognostic value of the SOCS/JAK2/STAT1/STAT3 axis for DN.

Insulin-like growth factor-1 (IGF-1), strongly regulating glomerular and tubular cells, is a growth factor for maintaining the nephritic structure and function and is an important player in DN pathogenesis [25]. Previous studies reveal that IGF-1 overexpression induces kidney tissue hyperplasia, renal cell proliferation, nephromegaly, mesangial expansion, and increased expression of inflammatory cytokines and ECM proteins [26]. IGF-1 is the ligand for the insulin-like growth factor-1 receptor (IGF-1R) [27]. IGF-1R inhibitor is suggested to decrease mesangial matrix and exapnsion in kidneys [28], alleviate inflammatory response and renal fibrosis [29,30] and stabilize podocyte integrity and reduce glomerular proteinuria in high-glucose-stimulated podocytes under diabetic conditions [31]. However, the biological functions of AG1024 (an IGF-1R inhibitor) in DN remain unknown. Hence, this study purposed to detect the biological functions of AG1024 and whether its functions were mediated by the SOCS/JAK2/STAT1/STAT3 axis in DN. We hypothesized that AG1024 would alleviate DN-like symptoms in animals. We believed that AG1024 may be an effective strategy for DN prevention.

## 2 Materials and methods

### 2.1 Experimental animals

A total of 60 Wistar rats (female, 8 weeks, 200–220 g) were purchased from the Jiangsu Aniphe Biolaboratory Inc and housed in a specific pathogen-free facility (55 ± 2% humidity, 21 ± 2°C, 12 h light/dark cycle) with free water and food.

### 2.2 Animal model and groupings

After 7 days of acclimation, the rats were randomly assigned into the control, the DN, the DN + AG1024, and the DN + metformin groups. Each group had 15 rats. The overnight-fasted rats were intraperitoneally injected with 2% pentobarbital sodium (40 mg/kg; Bio-techne, Shanghai, China) followed by the removal of the right kidneys. After 2 weeks, the rats in the DN groups received a single intraperitoneal injection of freshly prepared streptozotocin (STZ; 50 mg/kg) dissolved in ice-cold citrate buffer (10 mmol/L, pH 4.5) obtained from Sigma-Aldrich (Shanghai, China). In parallel, the rats were intraperitoneally injected with an equal volume of the citrate buffer without STZ. All animals were returned to their cages after complete recovery from anesthesia. After 72 h, blood samples were isolated from the tails to determine blood glucose levels, and the successful establishment criterion was a level >16.7 mmol/L. Insulin was not allowed in this study to avoid various effects of exogenous insulin.

The controls were injected once with citrate buffer and 0.5 mL saline daily for 12 weeks. After 4 weeks of cultivation, the diabetic rats in the DN group received 0.5 mL saline daily, those in the DN + AG1024 group were administered with AG1024 (20 mg/kg/day, *po*), and those in the DN + metformin group were given with metformin (200 mg/kg/day) by gavage for 8 weeks. AG1024 (purity 99.82%) and metformin (purity 99.64%) were purchased from MedChemExpress (Shanghai, China). The dosages of AG1024 and metformin were determined as previously documented [32,33].

### 2.3 Biochemical analyses

The body weight of each rat was monitored after drug administration. All rats were then housed in special metabolic cages for collecting 24 h urine, and the specimens were centrifuged at 3,000 rpm for 10 min at 4°C. The supernatant was used to measure the proteinuria concentration using ELISA. The collected blood samples from the right jugular artery were subjected to 10 min centrifugation at 3,000 rpm at 4°C, and the blood glucose, serum creatinine, and blood urea nitrogen concentrations were measured using commercially diagnostic available kits (Spinreact, Girona, Spain) by an enzymatic colorimetric method. After biochemical analyses, the rats were sacrificed by decapitation under anesthesia using 2% pentobarbital sodium (50 mg/kg), and renal tissue specimens were collected for subsequent use.

### 2.4 Histopathological examinations

The kidney tissues were fixed in 10% neutral buffered formalin for 24 h, paraffin-embedded, and sectioned into 4 μm-thick slices. The sections were stained with hematoxylin and eosin (H&E; Yuanye Biotechnology, Shanghai, China) and Masson’s trichrome (Solarbio, Beijing, China). The morphological changes were observed under a light microscope (Leica, Wetzlar, Germany).

### 2.5 ELISA

Measurement of inflammatory cytokines, including IL-6, MCP-1, IL-1β, and IGF-1 in renal tissues, was performed using specific ELISA kits (Boster Biotechnology, Wuhan, China).

### 2.6 Western blotting

Rat renal tissues were homogenized in 0.5 mL of radioimmunoprecipitation assay (RIPA) lysis buffer (Solarbio) for protein separation. The protein concentration of each sample was estimated using a BCA protein assay kit (Vazyme, Nanjing, China). For detection of protein levels, protein lysates (40 μg protein loaded per lane) for each group of tissues were separated by sodium dodecyl-sulfate polyacrylamide gel electrophoresis (SDS-PAGE) gels and transferred onto polyvinylidene fluoride (PVDF) membranes followed by blocking using 5% nonfat milk. After 1 h of blocking, the membrane was incubated overnight with primary antibodies against IGF-1R (ab182408, 1:1,000; Abcam), β-actin (ab115777, 1:200; Abcam), COX2 (ab179800, 1:2,500; Abcam), iNOS (ab178945, 1:1,000; Abcam), TGF-β (ab179695, 1:1,000; Abcam), collagen IV (ab6586, 1:500; Abcam), fibronectin (ab45688, 1:3,000; Abcam), p-JAK2 (ab32101, 1:2,000; Abcam), JAK2 (ab108596, 1:5,000; Abcam), p-STAT1 (ab109461, 1:5,000; Abcam), STAT1 (ab230428, 1:750; Abcam), p-STAT3 (ab32143, 1:5,000; Abcam), STAT3 (ab68153, 1:2,000; Abcam), SOCS1 (ab65989, 1 μg/mL; Abcam), and SOCS3 (ab280884, 1:1,000; Abcam) at 4°C. Then, the membrane was incubated with anti-rabbit (ab97051, 1:20,000; Abcam) at room temperature for 2 h. The proteins were visualized with an enhanced chemiluminescence detection kit (Beyotime, Shanghai, China).

### 2.7 Statistics analysis

All data were analyzed using GraphPad Prism 6.0 software (GraphPad Inc, San Diego, CA, USA) and expressed as mean ± standard deviation. Statistical analysis was performed using Student’s *t*-test or one-way analysis of variance followed by Tukey’s *post hoc* test. *P*-value < 0.05 was considered statistically significant.


**Ethical/Institutional review board approval:** The experimental protocols were granted approval by the Ethics Committee of The Sixth Hospital of Wuhan, Affiliated Hospital of Jianghan University, and were designed consistent with the National Institutes of Health Guidelines for the Care and Use of Laboratory Animals.

## 3 Results

### 3.1 AG-1024 attenuates the metabolic disorders in an experimental rat model of DN

**Figure 1:**
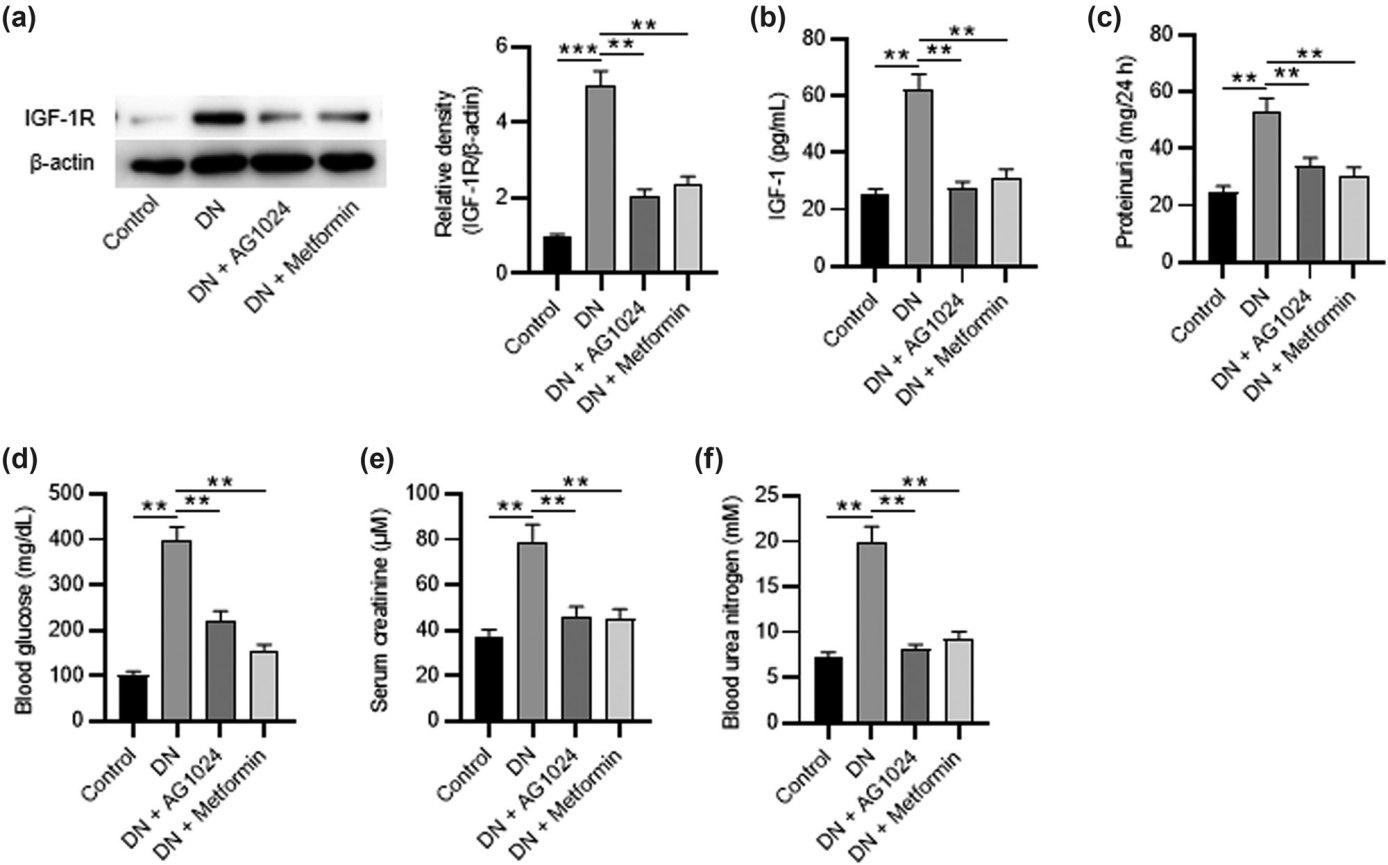
AG-1024 ameliorates metabolic disorders in rats with DN. (a) The protein levels of IGF-1R in renal tissues obtained from rats of the control, the DN, the DN + AG1024, and the DN + metformin groups were detected by western blotting. (b) ELISA of IGF-1 levels. (c) ELISA of 24-h proteinuria. (d) Blood glucose. (e) Serum creatinine. (f) Blood urea nitrogen. **p < 0.01 and ***p < 0.001. DN, diabetic nephropathy.

The renal tissues were collected 12 weeks after STZ injection, and the results of western blotting revealed that DN insult increased the protein level of IGF-1R, which was attenuated by AG1024 and metformin (Figure 1a). Then, the levels of IGF-1 in renal tissues from different groups were measured by ELISA, and the results revealed that the IGF-1 level was decreased in diabetic rats after administration of AG1024 or metformin (Figure 1b). Additionally, the promotion of 24 h proteinuria, blood glucose level, serum creatinine, and blood urea nitrogen in STZ-administered rats was abolished by AG1024. The positive control metformin had similar efficacy to AG1024 (Figure 1c–f). The above findings indicate that AG-1024 (an IGF-1R inhibitor) attenuates the metabolic disorders in the rat model of DN.

### 3.2 AG-1024 improves the renal function of diabetic rats

**Figure 2:**
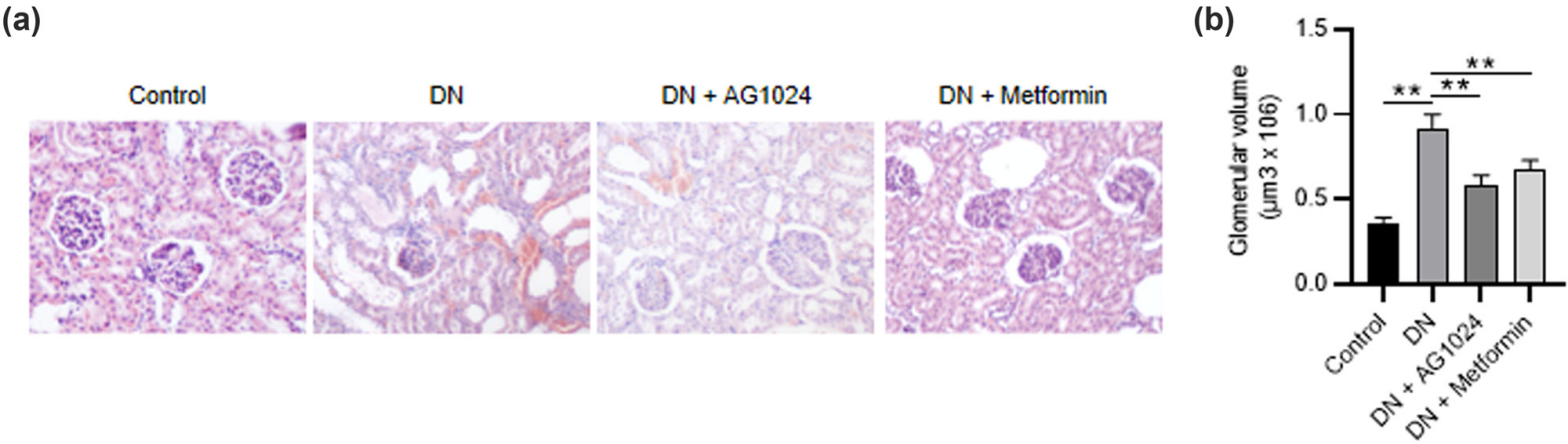
AG-1024 improves renal function in STZ-induced diabetic rats. (a) The H&E-stained renal tissues in the control, the DN, the DN + AG1024, and the DN + metformin groups (scale bar = 100 μm). (b) Quantification of glomerular volume. **p < 0.01. DN, diabetic nephropathy.

As revealed by H&E staining, the diabetic rats exhibited typical renal damage, including glomerular mesangial hyperplasia, ECM accumulation and expansion, glomerular sclerosis, atrophy, and inflammatory cell infiltration. After AG1024 administration, the abnormality of renal tissue morphology was alleviated, and the effect of AG1024 was similar to the positive control metformin (Figure 2a). Additionally, the diabetic rats showed decreased glomerular volume after administration of AG1024 or metformin (Figure 2b). Collectively, AG-1024 (an IGF-1R inhibitor) attenuates renal lesions in diabetic rats.

### 3.3 AG-1024 ameliorates renal inflammation in diabetic rats

**Figure 3:**
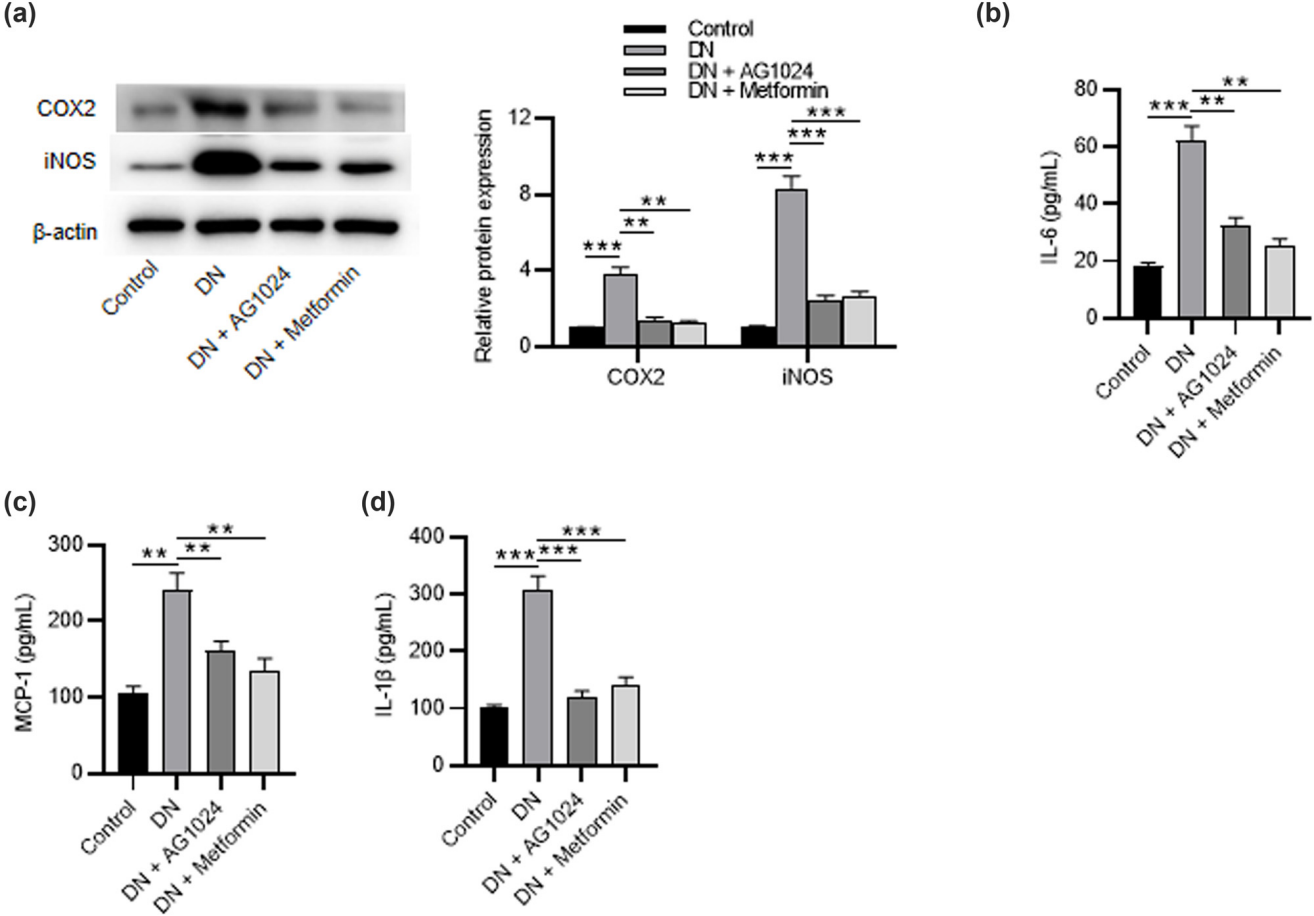
AG-1024 ameliorates renal inflammation in diabetic rats. (a) The protein levels of COX2 and iNOS in the renal tissues. ELISA of the (b) IL-6 level, (c) MCP-1 level, and (d) IL-1β level. **p < 0.01 and ***p < 0.001.

To explore the anti-inflammatory effects of AG-1024, we evaluated its effects on the expression of COX-2 and iNOS, key enzymes in the inflammatory processes of DN. The results of western blotting demonstrated that the expression of COX2 and iNOS in the kidneys of diabetic rats was significantly increased compared to the normal control group, while AG1024 or metformin administration attenuated the elevated expression of COX2 and iNOS (Figure 3a). Inflammatory cytokines are thought to play pivotal roles in the pathogenesis of DN. Cytokines including IL-6, MCP-1, and IL-1β are correlated with deterioration of the renal function [34]. We measured the expression of IL-6, MCP-1, and IL-1β in diabetic rat kidneys using ELISA. The results showed that administration of AG1024 or metformin abolished the DN-induced promotion in levels of inflammatory cytokines (Figure 3b–d). Taken together, the administration of AG-1024 (an IGF-1R inhibitor) attenuates renal inflammation.

### 3.4 AG-1024 reduces fibrosis formation in diabetic rats

**Figure 4:**
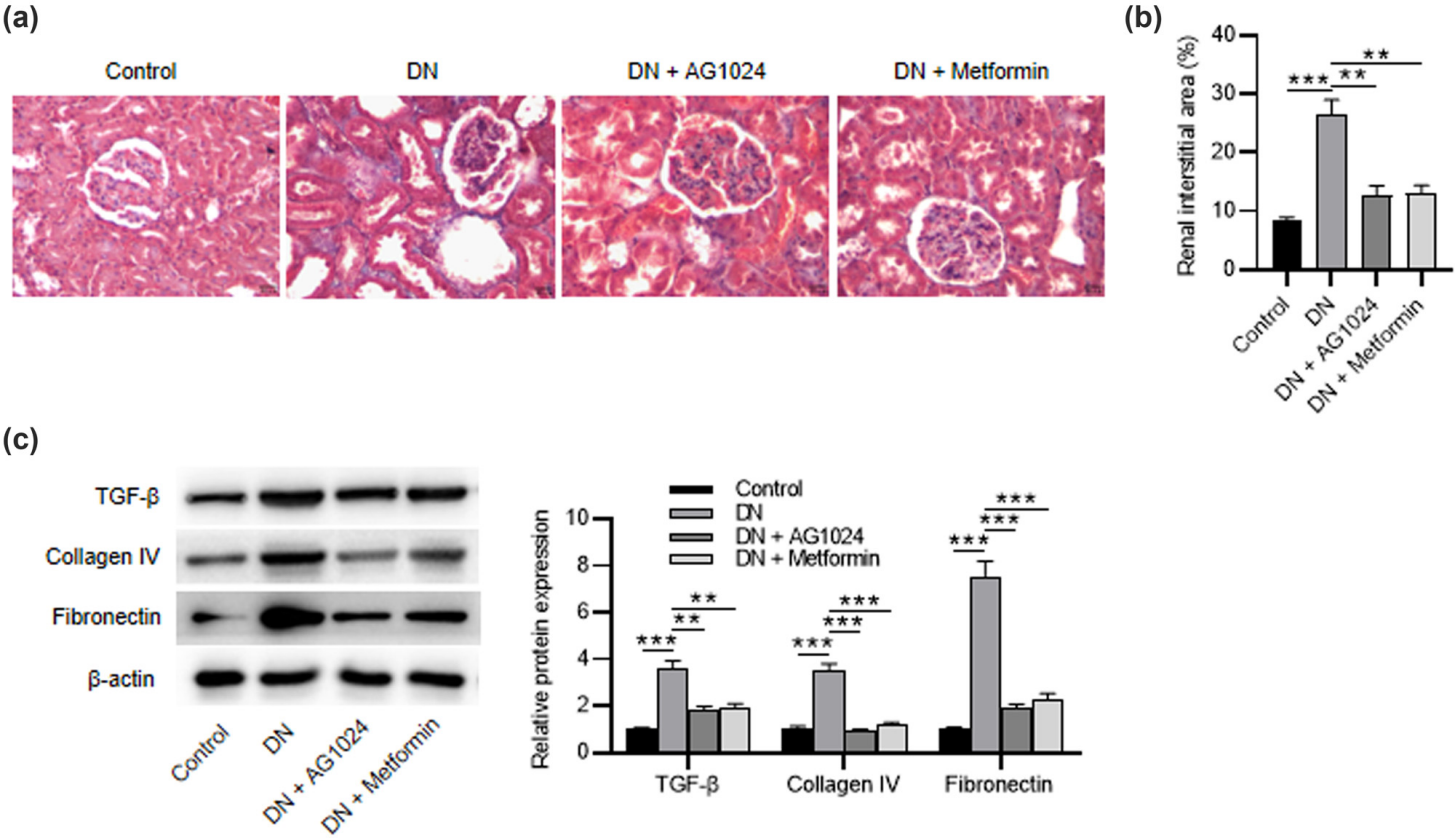
AG-1024 reduces fibrosis formation in diabetic rats. (a) Masson-stained renal tissues (scale bar = 100 μm). (b) Quantification of renal interstitial area. (c) The protein levels of TGF-β, collagen IV, and fibronectin. **p < 0.01 and ***p < 0.001.

The results of Masson staining revealed that the area of interstitial fibrosis was increased after DN induction. However, the administration of AG1024 or metformin has the opposite effects (Figure 4a and b). Then, the findings were confirmed via the western blotting method. The levels of fibrosis-associated proteins (TGF-β, collagen IV, and fibronectin) were decreased in diabetic rats after treatment with AG1024 or metformin (Figure 4c). Taken together, AG-1024 (an IGF-1R inhibitor) protects renal tissues from DN-induced fibrosis.

### 3.5 AG-1024 upregulates SOCS1/3 expression and inactivates the JAK/STAT pathway

**Figure 5:**
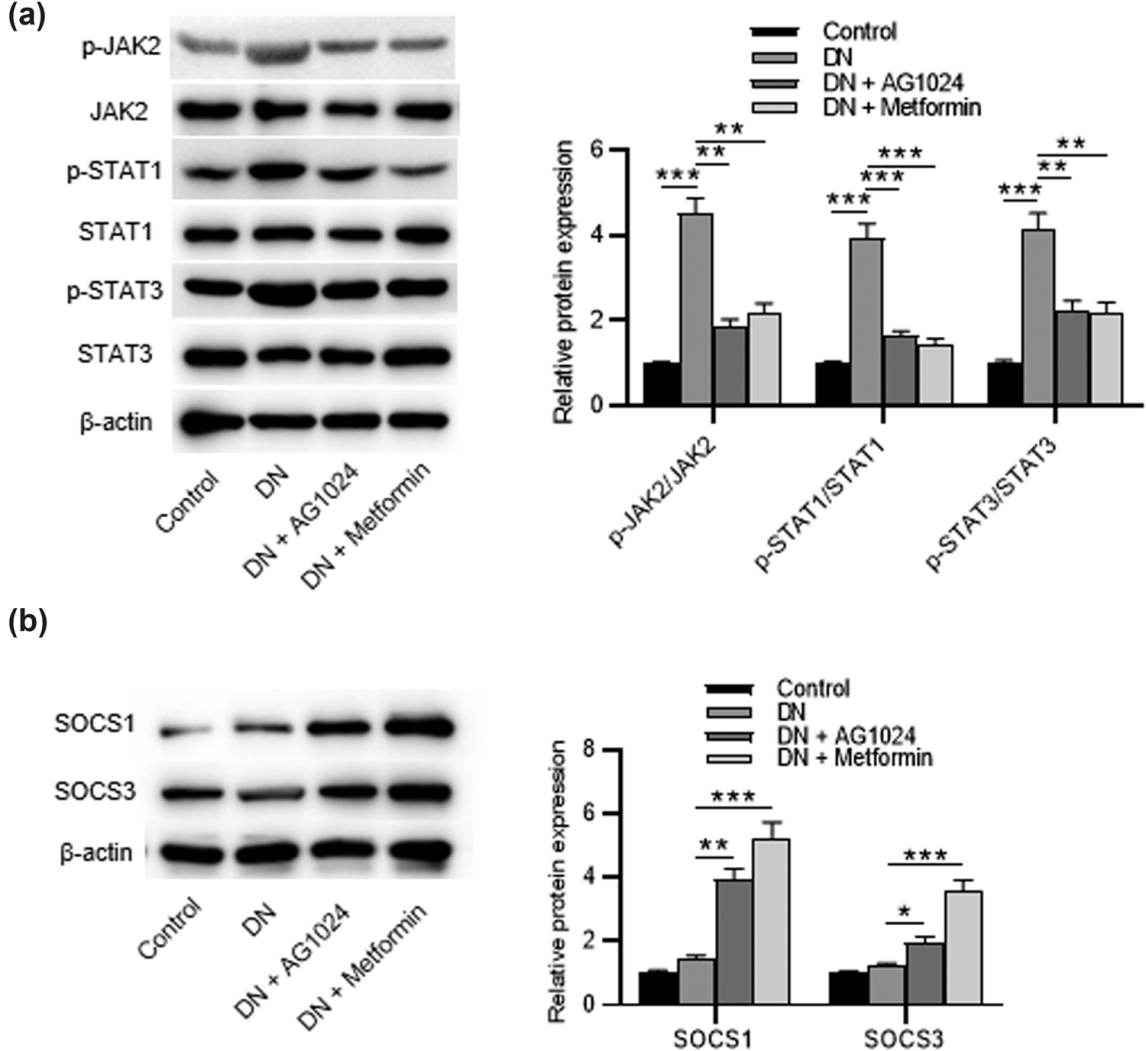
AG-1024 upregulates SOCS1/2 expression and inactivates the JAK2/STAT1/STAT3 axis. (a) The protein levels of phosphorylated JAK2, STAT1, and STAT3. (b) The protein levels of SOCS1 and SOCS3. * *p* < 0.05, **p < 0.01, and ***p < 0.001.

**Figure 6:**
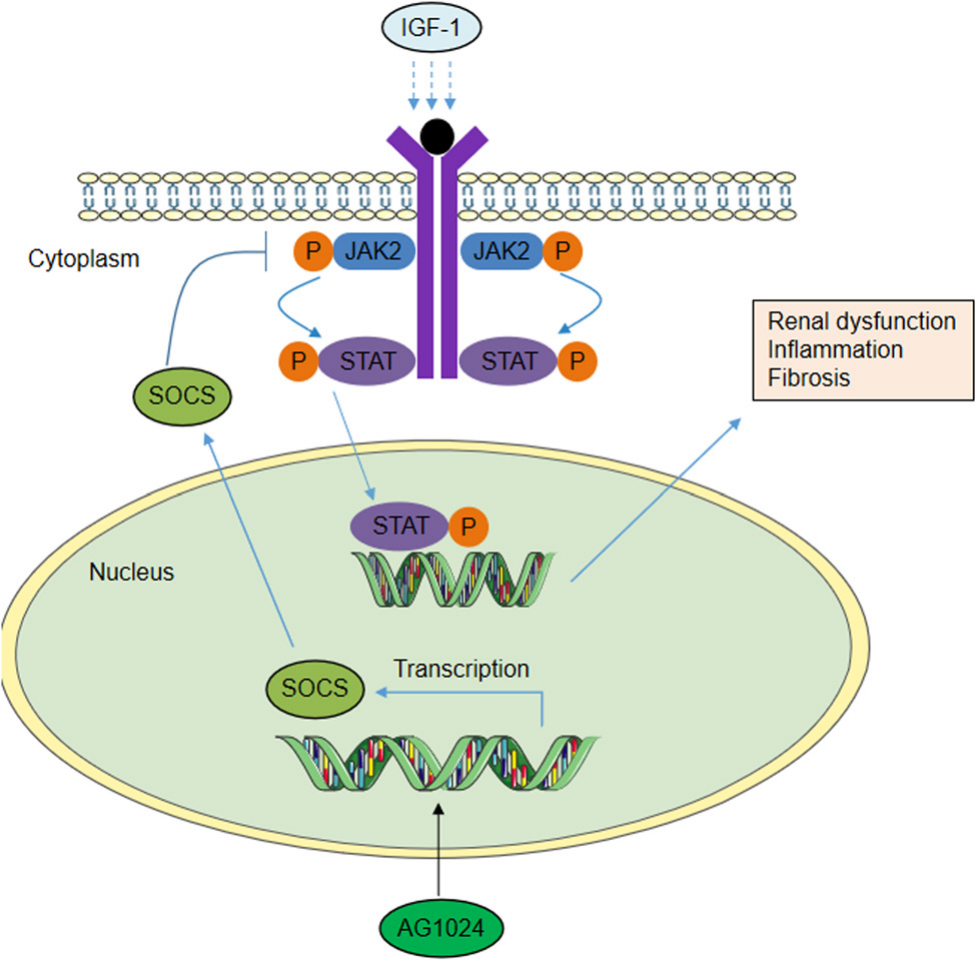
Schematic diagram depicting AG-1024 alleviates renal injury in experimental DN.

To investigate whether the protective effect of AG-1024 was related to the JAK2/STAT1/STAT3 axis, western blotting was performed to detect the levels of phosphorylated JAK2, phosphorylated STAT1, and phosphorylated STAT3. The results showed that AG1024 or metformin administration reduced the phosphorylation of JAK2, STAT1, and STAT3 in rats with DN (Figure 5a). To detect the mechanism of the JAK/STAT cascade, the protein levels of SOCS1 and SOCS3 were measured. The protein levels of SOCS1 and SOCS3 were significantly elevated by AG-1024 in diabetic rats, as shown by western blotting (Figure 5b). Figure 6 presents the scientific schematic diagram depicting the mechanisms by which AG1024 alleviates renal injury in experimental DN. In conclusion, AG-1024 upregulates the SOCS1/3 expression and inactivates the JAK2/STAT1/STAT3 axis.

## 4 Discussion

This study first demonstrated that IGF-1R was upregulated in renal tissues collected from experimental DN, and thereafter revealed the biological effects of its inhibitor (AG-1024) on metabolic parameters, renal morphological changes, renal inflammation, and renal fibrosis. Finally, we detected whether the protection of the IGF-1R inhibitor was associated with the JAK/STAT signaling and SOCS family.

Renal inflammation and fibrosis are critical players in DN pathogenesis and development. These two pathological processes are induced by profibrotic factors and inflammatory cytokines [16]. Glomerular inflammatory infiltration and progressive glomerulosclerosis facilitate podocyte demise and urinary albumin excretion in diabetic renal fibrosis [35]. AG1024 is found to attenuate inflammation and proliferation of neurotensin in colonic epithelial cells [36]. Additionally, other IGF-1R inhibitors, including GSK4529, can ameliorate diabetic kidney disease by amelioration of inflammatory infiltration and tubulointerstitial fibrosis by suppressing Snail1 expression [29]. Moreover, (−)-epigallocatechin gallate is found to attenuate chronic inflammation and improve hyperinsulinemia by blocking the IGF/IGF-1R axis [37], insulin deficiency induces rat renal mesangial cell dysfunction by activating IGF-1/IGF-1R signaling [38], and the anti-fibrotic effects of berberine are associated with decreased myocardial IGF-1R in diabetes [39]. In the current study, we found that AG1024 administration attenuated metabolic disorders and histopathological changes and decreased the levels of inflammatory cytokines, inducible enzymes, and fibrosis-associated proteins in DN rats, indicating that AG-1024 alleviates renal injury via attenuation of renal inflammation and fibrosis in diabetic rats.

The JAK/STAT pathway can regulate inflammation, proliferation, and the transmission of fibrotic responses [40]. Pharmacological modulation of this signaling has therapeutic potential in DN. Antidiabetic, cholesterol-lowering, and anti-hypertensive drugs are found to inactivate the JAK/STAT pathway during diabetes and mitigate renal inflammation in experimental DN [41]. AG1024 is found to decrease the expression of phosphorylated AKT in BCR-ABL-expressing cells and colonic epithelial cells [36,42], and also has been suggested to downregulate the expression of phosphorylated AKT and STAT3 in DU145 cells [43]. The current study showed that the administration of AG1024 inhibited the phosphorylation of JAK2, STAT1, and STAT3 in diabetic rats. Studies have recognized SOCS expression as an endogenous mechanism to regulate JAK/STAT overactivation in renal diseases [23,24]. As reported, SOCS proteins can negatively mediate the JAK/STAT pathway [44]. Delivery of SOCS1-3 proteins in experimental models of DN can mitigate the pathological features of DN [45,46]. Here, we found that protein levels of SOCS1 and SOCS3 were significantly upregulated by AG1024. In conclusion, AG1024, an IGF-1R inhibitor, ameliorates renal injury by attenuating renal inflammation and fibrosis by the SOCS/JAK/STAT pathway.

There are limitations to this study. First, studies on the specific functions of this pathway are lacking. Further experiments are required to investigate the functions of the overexpressed or blocked pathway in the context of AG-1024. Second, clinical data of patients are lacking. Third, experiments were performed at a single time point; additional time points also require investigation. Despite these limitations, we believe that AG1024 may be promising for DN prevention.
